# Rettigheter og tvang i kommunale hjelpetilbud til personer med alvorlige samtidige rus- og psykiske lidelser: erfaringer med regelverket, i lys av menneskerettigheter

**DOI:** 10.1177/14550725231156479

**Published:** 2023-06-26

**Authors:** Ane Bendixen, Hildegunn Sagvaag, Bjørn Henning Østenstad, Trond Grønnestad

**Affiliations:** 56627 University of Stavanger, Stavanger, Norway; 56627 University of Stavanger, Stavanger, Norway; 1658 University of Bergen, Bergen, Norway; 56627 University of Stavanger, Stavanger, Norway

**Keywords:** human rights, legal framework, municipal services, substance use disorders, mental illness, co-occurring disorders (COD), qualitative research

## Abstract

Rights and coercion in municipal services for persons with co-occurring severe mental illness and substance use disorders – experiences with legal framework, in light of human rights. **Aims:** Persons with co-occurring severe mental illness and substance use disorders can have a considerable need for municipal health and care services but can be difficult to reach with such services. In Norway, there are known perceived ambiguities and obscurities in the legal framework for such services. This study aims to further examine these legal issues in a Norwegian context, by examining what challenges service providers experience in the practice of the current legal framework in this field. **Design:** The data consists of 13 qualitative interviews with strategically selected service providers from Norwegian municipalities and county governors’ offices. The interviews were analyzed through systematic text condensation. **Results:** The participants reflections resulted in three especially salient perceived challenges in the practice of the current legal framework in this field: ‘being dependent upon extra efforts that exceed the legal minimum requirements’, ‘lacking a legal basis and tools’ and ‘a complex and composite legal framework’. **Conclusions:** When seen in light of human rights, the three identified challenges in legal framework should be considered more closely both from a research perspective and from a policy making perspective. It should be investigated further whether human rights oblige the state beyond setting forth minimum requirements, how different human rights impact one another, especially with a view to service providers’ rights v. service recipients’ rights, and lastly if it is feasible to simplify or clarify the current legal framework to ensure adherence to the law and to promote equal practice among service providers.

## Bakgrunn

Personer med alvorlige samtidige rus- og psykiske lidelser (ROP-lidelser) kan ha et stort behov for helse- og omsorgstjenester, men vanskeligheter med å nyttiggjøre seg tilbud. I denne studien har vi utforsket utfordringer i det norske regelverket for kommunale bo- og tjenestetilbud til denne gruppen, sett fra et tjenesteyterperspektiv. Studien tar utgangspunkt i den norske konteksten, men kan ha overføringsverdi til resten av Norden og andre velferdsstater. Lignende tematikk som studien tar utgangspunkt i er for eksempel skissert i både Danmark (Bjerge et al., 2020; Brasch et al., 2011; Nygaard-Christensen et al., 2018) og Sverige (Matscheck & Piuva, 2022; Socialstyrelsen, 2019; Statens offentliga utredningar, 2021).

Rus- og psykiske lidelser «påvirker hverandre gjensidig og krever spesiell oppmerksomhet» (Helsedirektoratet, 2012, s. 3). Personer med rus- og/eller psykiske lidelser er i økende grad en prioritert gruppe i offentlige strategier og tiltak (se Prop 92 L (2020–2021) for en oversikt over tiltak av nyere dato). Alvorlighetsgraden av lidelser samt tjenestebehov hos personer med ROP-lidelser er svært varierende (Helsedirektoratet, 2014). Personer med *alvorlige* ROP-lidelser kan ha en vesentlig nedsatt funksjonsevne, og dermed behov for ulike tjenester og ytelser fra kommunens helse- og omsorgstjeneste, NAV (Arbeids- og velferdsforvaltningen) og spesialisthelsetjenesten, og kan i tillegg ha et forhold til politi og kriminalomsorgen (NOU 2019: 14).

Kommunene har over tid fått et stadig større ansvar for tjenestetilbudet til denne gruppen (Kommunal- og moderniseringsdepartementet, 2020), som kan ha behov for både bo- og tjenestetilbud fra kommunens helse- og omsorgstjeneste. Blant de som er tildelt kommunal bolig utgjør personer med ROP-lidelser en økende andel (Solstad et al., 2021). Deres tjenestebehov kan være krevende å imøtekomme, «de faller lett utenfor behandlingstiltak» (Helsedirektoratet, 2012, s. 13), og kommunene «opplever ofte arbeidet som svært krevende» (Kommunal- og moderniseringsdepartementet, 2020, s. 19). Blant annet er personer med ROP-lidelser overrepresentert blant bostedsløse (Solstad et al., 2021). Personer med alvorlige ROP-lidelser kan altså ha åpenbare behov for kommunale bo- og tjenestetilbud, men kan være vanskelige å nå og å etablere tilpassede tilbud for.

Kommunens bo- og tjenestetilbud til personer med alvorlige ROP-lidelser reguleres av helselovgivningen som består av regler om virksomhetens plikter (helse- og omsorgstjenesteloven, 2011), det enkelte helsepersonells plikter (helsepersonelloven, 1999), og tjenestemottakerens rettigheter (pasient- og brukerrettighetsloven, 1999). I tillegg reguleres leieforhold av husleieloven (1999). Gruppens behov for sammensatte tjenester (Helsedirektoratet, 2014), gjør det videre viktig å se sammenhenger med sosiallovgivningen og reguleringen av spesialisthelsetjenesten. Helselovgivningen og husleieloven (1999) regulerer til sammen personer med ROP-lidelser sin rett til å motta, og virksomhetens og helsepersonellets plikt til å yte *tjenester*. Videre reguleres til dels *organiseringen* av disse tjenestene. I tillegg reguleres rett til selvbestemmelse og til dels grensene for denne retten, gjennom rammer for inngrep som kan være aktuelle i enkelte tilfeller. Reglene er generelle eller har andre kjernemålgrupper enn personer med alvorlige ROP-lidelser, og det finnes ingen egen regulering for det kommunale helse- og omsorgstilbudet til gruppen. Det rettslige rammeverket for kommunale botilbud har av Nasjonalt kompetansesenter for psykisk helsearbeid (NAPHA) blitt beskrevet slik: det kan «stilles store spørsmål ved beboernes rettsikkerhet: de juridiske uklarhetene er åpenbare» (NAPHA, 2017, s. 48). Et behov for å «klargjøre de juridiske rammene» som gjelder botilbud til personer med ROP-lidelser er også uttrykkelig fremhevet i den nyeste nasjonale strategien for den sosiale boligpolitikken (Kommunal- og moderniseringsdepartementet, 2020, s. 19).

De tradisjonelle, nasjonale lovreglene må være i samsvar med grunnleggende menneskerettighetsbestemmelser staten er bundet av. Disse utgjør essensielle garantier i en rettstat. Den praktiske nytten og gjennomslagskraften av menneskerettighetene vil være særlig stor på områder hvor nasjonal rett er uklar eller utilstrekkelig, slik dette rettsområdet er beskrevet å være. Det gjelder også når reglene omhandler utsatte grupper i samfunnet, slik som personer med alvorlige ROP-lidelser, som må antas å ha en lavere grad av rettssikkerhet enn majoriteten i befolkningen (NAPHA, 2017).

Kommunale bo- og tjenestetilbud skal ivareta flere sentrale menneskerettigheter for personer med alvorlige ROP-lidelser. Tilbudet skal blant annet ivareta personenes rett til høyest oppnåelig helsestandard både fysisk og psykisk (FNs konvensjon om økonomiske, sosiale og kulturelle rettigheter [ØSK], 1966, art. 12), retten til en tilfredsstillende levestandard, inkludert en tilfredsstillende bolig (ØSK, 1966, art. 11), samt personenes rett til selvbestemmelse, respekt for sin integritet og privatliv (Den europeiske menneskerettskonvensjon [EMK], 1950, art. 8; Grunnloven, 1814, § 102). Disse rettighetene kan også i enkelte tilfeller komme i et motsetningsforhold til hverandre, for eksempel når personer motsetter seg helsehjelp og dermed foretar valg som ikke er til det beste for egen helse. De nevnte menneskerettighetene, og avveiningene mellom dem, kan finnes igjen i helsetjenestens verdigrunnlag, og i diverse lovverk,for eksempel gjennom retten til nødvendige helse- og omsorgstjenester i pasient og brukerrettighetsloven (1999) § 2-1 a, og i reglene om samtykke til helsehjelp i pasient- og brukerrettighetsloven (1999) kapittel 4. Sentrale menneskerettighetsdokumenter er også gjort til norsk lov gjennom menneskerettsloven (1999).

Menneskerettighetene gir den norske stat forpliktelser overfor sine borgere. Staten fritas ikke fra dette ansvaret ved å delegere oppgaver til kommunene (Eide, 2006). Staten skal sikre rettighetene - respektere, beskytte og oppfylle rettighetene - ved å avstå fra å selv gjøre inngrep i dem, ved å beskytte personer mot brudd på rettighetene fra andre personer, og ved å gjøre nødvendige, aktive tiltak for å sikre at det ytes velferdstjenester som ivaretar rettighetene (Eide, 2006; Kjellevold & Aasen, 2011; UN Committee on Economic, Social and Cultural Rights [CESCR], 2000). For eksempel kan ikke staten garantere for at alle innbyggere har god helse og aldri opplever sykdom eller skade, men den er forpliktet til å sørge for at det finnes et fungerende helsevesen, med bestemte krav til dette systemet (Eide, 2006; CESCR, 2000). I denne artikkelen har vi derfor valgt å bruke begrepet ‘retten til tjenester’, som fellesbetegnelse på retten til helse og tilfredsstillende levestandard inkludert bolig. Staten kan heller ikke garantere for at alle innbyggere til enhver tid kan ha en absolutt selvbestemmelse eller absolutt privatliv, så denne rettigheten kan det på nærmere vilkår gjøres unntak fra, når andre viktige hensyn gjør seg gjeldende (EMK, 1950, art. 8, andre ledd). Statens plikt til å sikre menneskerettighetene gjelder overfor personer med ROP-lidelser på lik linje med resten av befolkningen (Grunnloven, 1814, § 98; ØSK, 1966, art. 2).

Det foreligger lite forskning på de rettslige rammene for kommunale bo- og tjenestetilbud til personer med alvorlige ROP-lidelser. I en rapport fra 2017 ser NAPHA nærmere på bruk av rettighetsinngripende tiltak i kommunale botilbud, og peker på noen problemstillinger som erfares å ha stor praktisk betydning av ansatte i slike boliger (NAPHA, 2017; se også Kommunal- og moderniseringsdepartementet, 2020). Eksempler er begrensninger knyttet til besøk, og i hvilke tilfeller kommunen kan ta seg inn i privat bolig. Rapporten til NAPHA knytter i begrenset grad disse problemstillingene opp til gjeldende lovverk, men henviser i stedet til det da pågående arbeidet til Tvangslovutvalget (NAPHA, 2017). Tvangslovutvalget (NOU 2019: 14) gir heller ikke en slik inngående behandling av disse problemstillingene som rapporten fra NAPHA ser ut til å forutsette. Tvangslovutvalget (NOU 2019: 14) behandler i hovedsak tvang i institusjon og ved ytelse av helse- og omsorgstjenester uavhengig av arena, og tar i liten grad opp selve bostedsproblematikken som sådan. Selv om husleielovens (1999) regler nevnes noen steder, er ikke situasjonen spesifikt for personer med ROP-lidelser og implikasjonen av lovforslaget i denne retning, i nevneverdig grad omtalt. Det andre større lovarbeidet av nyere dato med stor betydning for personer med alvorlige ROP-lidelser, Rusreformen (NOU 2019: 26; se også Prop 92 L (2020–2021)), går heller ikke i dybden på bostedsproblematikken. Menneskerettighetenes betydning for kommunale tilbud til personer med ROP-lidelser er også i liten grad behandlet i litteratur og forskning. Diskusjoner rundt menneskerettighetene på helse- og omsorgsfeltet har særlig knyttet seg til rammene for tvangsbruk, og da først og fremst for andre grupper og arenaer enn kommunale bo- og tjenestetilbud til personer med alvorlige ROP-lidelser, for eksempel personer med psykisk utviklingshemning og reglene i psykisk helsevern (Østenstad, 2018).

Det foreligger altså tydelige kunnskapshull knyttet til hvilke deler av regelverket som oppleves problematisk eller uklart i praksis. På denne bakgrunn ønsket denne studien å utforske problemstillingen:

Hvilke utfordringer erfares i regelverket for kommunale bo- og tjenestetilbud til personer med alvorlige ROP-lidelser fra et tjenesteyterperspektiv?

Det foreligger også tydelige kunnskapshull knyttet til menneskerettighetenes betydning for dette regelverket. Derfor vil vi drøfte funnene fra studien i lys av grunnleggende menneskerettigheter.

## Metode

### Studiens design

For å kunne utforske hvilke utfordringer i rettslig rammeverk tjenesteyterne har erfart i organiseringen og gjennomføringen av kommunale bo- og tjenestetilbud til personer med alvorlige ROP-lidelser, fremstod en kvalitativ, eksplorativ studie med semi-strukturerte intervjuer som relevant (Kvale & Brinkmann, 2015). Studien ble gjennomført av førsteforfatter, som er jurist. Målet har vært å avdekke faktiske utfordringer *i møtet mellom* rettslig rammeverk og praksis, heller enn å kartlegge praksis sin *kunnskap om regelverket som sådan*. Intervjuene har derfor vært gjennomført med deltakere med størst mulig praktisk erfaring med de relevante rettslige rammene og tjenestetilbudet. Intervjuene har gitt kunnskap om deltakernes opplevelse av sin praktiske virkelighet, og hvor de har opplevd at rettslig rammeverk har hemmet, eller i det minste ikke fremmet, gode tjenester.

### Deltakere og rekruttering

Det ble rekruttert to deltakere fra hver av de fire ulike kommunene i studien. To deltakere fra kommunene var jurister, og samtlige deltakere arbeidet som rådgivere, enhetsleder o.l. som gjør at de var blant personene i kommunen med størst kunnskap om de relevante temaene, i kommuner med tilstrekkelig størrelse til å ha tilpassede tilbud til personer med alvorlige ROP-lidelser. Ansatte hos statsforvaltere fra fylkene hvor kommunene ligger ble videre rekruttert, totalt fem ansatte hos tre ulike statsforvaltere, hvorav fire var jurister. For å få tak i personer med særlig kunnskap om hvordan rettslig rammeverk erfares å fungere i praksis ble det gjort et strategisk utvalg (Malterud, 2017; Patton, 2015). Målet med studien var altså ikke å oppnå representativitet, men å få dybdekunnskap om utfordringer tjenesteyterne erfarte. Rekrutteringen av deltakere ble gjort gjennom en kombinasjon av åpen forespørsel som ble delt i kommune-nettverk, forskningsforbindelser, snøball-rekruttering (Waters, 2015) og direkte henvendelser til personer som var aktuelle som deltakere.

### Datainnsamling

For å sikre relevante data ble det utarbeidet en semi-strukturert intervjuguide (Kvale & Brinkmann, 2015). Intervjuguiden inneholdt åpne spørsmål om blant annet organisering av tjenestene og rettighetsbegrensninger i privat bolig, samt generelle spørsmål rundt eventuelle utfordringer deltakerne erfarte, inkludert hvilke endringer de mente burde gjøres i lovverket. Innholdet i intervjuguiden bygget på problemstilling og tilgjengelig litteratur inklusive rettslige kilder (helse- og omsorgstjenesteloven, 2011; Helsedirektoratet, 2012, 2014; husleieloven, 1999; Kommunal- og moderniseringsdepartementet, 2020; NAPHA, 2017; NOU 2019: 14; pasient- og brukerrettighetsloven, 1999; Østenstad, 2011; Søvig, 2007). Førsteforfatter gjennomførte 11 individuelle intervjuer og ett intervju med to deltakere (etter informantenes ønske), over telefon tidlig våren 2022. Telefonintervju ble valg på grunn av koronasituasjonen og av ressurshensyn. Ved telefonintervjuene var det ikke mulig å oppfatte ikke-verbal kommunikasjon, og gjennomføringen var avhengig av gode tekniske løsninger, men telefonintervju muliggjorde enklere tilgang på et større geografisk område, og anses ikke for å være vesentlig dårligere enn vanlig intervju (Cachia & Millward, 2011; Holt, 2010; Svensson et al., 2020).

Intervjuene ble innledet med en presisering av at målet med studien var å se på både utfordringer, muligheter og begrensninger i regelverkets møte med praksis, og at fokus var på både hva som fungerte og hva som ikke fungerte. Alle intervjuer ble tatt opp digitalt. Intervjuene varte mellom 38 og 67 minutter og var gjennomsnittlig på 51 minutter. Etter intervjuer med 13 deltakere ble dataene ansett for å være rikholdige nok til å kunne gi viktig informasjon om forskningsspørsmålet (Malterud, 2017).

### Forskningsetikk

Deltakerne fikk muntlig og skriftlig informasjon om studien før intervju ble gjennomført, og deltakerne signerte skriftlig samtykkeerklæring. Det ble gjort klart at informantene til enhver tid kunne trekke seg fra studien uten at det ville få konsekvenser for dem. Studien ble godkjent av Regionale komiteer for medisinsk og helsefaglig forskningsetikk (REK), som del av en større studie (saksnr. 202928).

### Dataanalyse

Lydfilene fra intervjuene ble transkribert og analysert ved hjelp av systematisk tekstkondensering, slik denne beskrives av Malterud (2017). Analysen besto av flere steg. Forfatterne leste et utvalg intervjuer for å identifisere foreløpige temaer på tvers av intervjuene, som ble diskutert i forskningsteamet og videreutviklet til kodegrupper. Meningsenheter innen de identifiserte kodegruppene ble organisert i subgrupper, som igjen ble diskutert mellom forfatterne. Innholdet i subgruppene ble så kondensert i kunstige sitater og det ble trukket ut såkalte gullsitater, i tråd med Malterud (2017). Til slutt ble det laget synteser, altså analytiske tekster, til hver av kodegruppene og subgruppene. Førsteforfatter har kontinuerlig gått tilbake til de opprinnelige transkriberte intervjuene og arbeidet refleksivt (Alvesson & Skõldberg, 2018; Malterud, 2017), og videreutviklet kodegruppene og subgruppene. Blant annet var de opprinnelige teamene nærere knyttet til temaene i intervjuguiden, for eksempel i temaet «Organisering», men disse ble videreutviklet til kodegrupper som tydeligere presiserte hva slags type utfordring i lovverket det var tale om.

## Resultater

Resultatene fra undersøkelsen kan deles inn i tre hovedfunn, altså tre hovedutfordringer som deltakerne beskrev i de rettslige rammene for kommunale bo- og tjenestetilbud til personer med alvorlige ROP-lidelser. For det første beskrev deltakerne store variasjoner i tjenestetilbudet, og en opplevelse av **å være avhengig av ‘ekstrainnsats’ ut over lovverkets minimumskrav**. Den andre utfordringen var at deltakerne opplevde at de **manglet inngrepshjemler og/eller verktøy**, for å ivareta de dårligste og de farligste tjenestemottakerne, også på bekostning av egne ansatte. Den siste utfordringen var at deltakerne opplevde det gjeldende rettslige rammeverket som **komplisert og sammensatt**. De tre hovedfunnene kan deles inn i følgende delfunn:

1. En opplevelse av å være avhengig av ‘ekstrainnsats’ ut over lovverkets minimumskrav
  - Beslutningsstøtte og veiledning
  - ‘Masing’ og å ikke gi opp
  - Forskjeller i botilbud
2. En opplevelse av manglende hjemler og verktøy
  - De ‘dårligste og svakeste’
  - De ‘farlige’
  - Beskyttelse av arbeidsmiljø
3. En opplevelse av et komplisert og sammensatt regelverk
  - Generell kompleksitet
  - Forvirring rundt organiseringen av botilbud

I intervjuene kom det også frem refleksjoner rundt mulige løsninger på utfordringene. For eksempel var det delte meninger rundt om tvang burde kunne benyttes i kommunen i større omfang, eller være forbeholdt spesialisthelsetjenesten. Fra statsforvalterne var det også ulike synspunkt på om utfordringene delvis kunne løses gjennom det som kanskje kan kalles utradisjonelle tolkninger av gjeldende rett, for eksempel ved bruk av ulovfestede rettsgrunnlag og analogier fra andre regelsett, eller om det trengtes endringer i det aktuelle regelverket. Deltakerne skisserte også hvordan de hadde løst problemstillinger som andre steder har blitt framholdt som utfordringer (NAPHA, 2017), for eksempel knyttet til husordensregler og samarbeidsavtaler. Ettersom denne artikkelen omhandler utfordringene deltakerne erfarte i regelverket, avgrenses det mot en nærmere redegjørelse for andre deler av deltakernes refleksjoner.

### En opplevelse av å være avhengig av ‘ekstrainnsats’ ut over lovverkets minimumskrav

De fleste deltakerne beskrev at det etter deres oppfatning ble ytt tjenester ut over det lovverket krevde, og at dette var helt nødvendig for å lykkes med et godt tjenestetilbud til personer med alvorlige ROP-lidelser. Flere beskrev at ansatte forsøkte å rettlede i velferdssystemet, at de forsøkte å finne kreative løsninger innenfor lovverket, og at de ønsket seg gode, tilrettelagte botilbud. Samtidig varierte det hvor godt deltakerne beskrev at ekstrainnsatsen fungerte, og tilbudet og synet på ekstrainnsatsen syntes å variere mellom kommuner. Noen deltakere beskrev at man var fullt ut avhengig av et godt sykepleiefaglig og miljøfaglig arbeid, at man var veldig avhengig av hvem tjenestemottakeren hadde kontakt med i hjelpeapparatet, og at tilbudet kunne være begrenset av politiske beslutninger som ikke hadde støtte i fagmiljøet.

Kommunen skal jo ikke gi optimale tjenester, de skal gi forsvarlige. Så lenge de er innenfor forsvarlige så er det innenfor lovverket. Det er kanskje litt av utfordringen til denne brukergruppen. Ofte så må du gjerne gi det lille ekstra for at det skal fungere. Hvis ikke så faller det veldig fort sammen. (Deltaker 9, statsforvalter).

De fleste deltakerne fremholdt at man måtte yte det lille ekstra overfor denne gruppen for at det skulle fungere, ved å «tåle» å jobbe systematisk over tid, finne de kreative løsningene innenfor lovverket, og ikke ta et nei for et nei til en viss grad. Noen deltakere fra statsforvalterne fremhevet at tjenesteyter kunne være nervøse for at dette gled over i tvang og restriksjoner, men at det både var tillatt og viktig å stå på og jobbe over tid. De fleste deltakerne fremholdt at det ble ytt mer enn det som sto i vedtakene. De beskrev at det var helt nødvendig å anstrenge seg mer enn lovverket krevde for å lykkes.

Satt på spissen, hvis man tenker kun husleieloven, så skulle disse bare fått, «Her har du en bolig, OK du ønsker ikke booppfølgingstjeneste, nei det er frivillig, OK da får du ikke det». Og litt sånn lykke til. (Deltaker 7, kommune).

I tillegg til innsatsen som var direkte knyttet til tjenesteytelsen, beskrev flere deltakere ekstrainnsats som knyttet seg til velferdssystemet som sådan, for eksempel hjelp med søknader til NAV, som blant annet har ansvar for trygdeytelser. De fleste deltakerne oppfattet dette som en ren praktisk løsning, eller at innsatsen skyldes faglige vurderinger. Deltakerne fremhevet at det egentlig var den aktuelle tjenesten, for eksempel NAV, som skulle bistå med veiledning. Én deltaker mente veiledning knyttet til andre velferdstjenester var en rettslig forpliktelse. Det ble fremhevet, særlig av deltakerne fra statsforvalterne, at det nok var svært varierende hvor mye kunnskap ansatte hadde om andre deler av velferdssystemet. En deltaker fortalte om tjenestemottakere med alvorlige dobbeltdiagnoser som ikke hadde uføretrygd. En annen deltaker fremhevet at mange tjenestemottakere hadde sagt opp vergen sin fordi de måtte betale egenandel. Det ble fremhevet, igjen særlig av statsforvalterne, at tjenestemottaker var prisgitt hvem de hadde kontakt med i systemet.

Det tror jeg også er noe med hele tjenestene. Rettsikkerheten for den gruppen. Det er jo en gruppe som ikke har så mange som står opp for seg. Veldig avhengig av hvem man har kontakt med i hjelpeapparatet. Som på en måte har den systemforståelsen for hvordan man skal gripe an å få dekket det behovet eller få de rettighetene de har krav på. (Deltaker 9, statsforvalter)

Gjennom deltakernes beskrivelser kom det også frem en omfattende variasjon i eksisterende botilbud, i hva man la vekt på i valget av tilbud, og i deltakernes holdninger til eller oppfattelse av de ulike tilbudene. I beskrivelsene av tilbud varierte det om og i hvilken grad kommunene hadde egne institusjonsplasser for gruppen, kjøpte botjenester fra private leverandører (såkalte «kjøpsplasser»), hadde bemannede og samlokaliserte boliger, eller individuelle boliger med ambulerende tjenester. «Noen kjøper nesten bare institusjonsplasser på rammeavtale, andre har kun vanlig kommunal bolig, noen har en bemannet bolig» (Deltaker 10, statsforvalter). Flere fortalte at de skulle ønske deres kommune hadde mer av noen tilbud og mindre av andre, samt mer tilpassede og differensierte tilbud. Noen deltakere var ikke fornøyde med egen kommune sitt tilbud, beskrev at det var vanskelig å finne gode botilbud, og sa at denne gruppen ble kasteballer i systemet. «Kommunen har egentlig ikke det rette tilbudet til de tenker jeg ofte» (Deltaker 4, kommune). En statsforvalter fremholdt at det var problematisk at organiseringen av tilbudet falt inn under kommunens frie skjønn så lenge tilbudet var forsvarlig, fordi for eksempel det å ha fast bemanning eller ha et høyere omsorgsnivå kunne ha avgjørende betydning for tjenestemottakeren. Noen deltakere uttrykket også frustrasjon rundt politiske føringer for organisering av botilbud, i forhold til blant annet størrelse på enheter og hva kommunen skulle kjøpe av private leverandører. Flere mente at de politiske føringene bygget på ressurshensyn. «Tydeligvis så beslutter de ting uten å rådføre seg med folk som jobber med brukergruppen. […] Disse politiske føringene kompliserer bildet av og til.» (Deltaker 11, kommune).

### En opplevelse av manglende hjemler og verktøy

Flere deltakere la vekt på at de vurderte at regelverket var lite utbygd med tanke på å kunne gripe inn overfor de dårligste, svakeste og/eller farligste tjenestemottakerne. For de dårligste og svakeste handlet det i stor grad om å klare å gi hjelp, mens det for de farlige også handlet om behov for kontroll og skjerming av omverdenen og egne ansatte. Noen uttrykket frustrasjon over at lovverket ikke var konsekvent, fordi deltakerne vurderte det som mye mer utbygd for personer som hadde en psykisk utviklingshemning eller somatiske helseproblemer. Deltakerne mente man hadde et lovtomt rom hvor man risikerte at de dårligste og svakeste kunne skade seg selv, og hvor man risikerte at de farligste kunne skade andre.

De utgjør jo en liten gruppe, men det er disse som faller mellom alle stoler egentlig. De er for kort tid inne i lukkede institusjoner, de ruser seg for mye, de samhandler lite med omgivelsene rundt seg, de er i utgangspunktet ikke interessert i å motta hverken tjenester eller sette seg inn i egen situasjon, og utsetter seg selv og andre for kontinuerlig belastning eller risiko. (Deltaker 2, kommune)

Flere deltakere opplevde det som frustrerende å ikke kunne skjerme ressurssvake personer fra skadelige miljøer, å ikke kunne gå inn i privat bolig uten samtykke, og å prøve å gi tjenester til personer som var så dårlige at relasjonelt arbeid ikke fungerte. Disse deltakerne uttrykket frustrasjon og bekymring over at de dårligste og svakeste tjenestemottakerne fikk «gå til grunne» uten innblanding. De opplevde at man måtte la tjenestemottakeren fortsette å skade seg selv, til det gikk så langt at man sto i en nødrettssituasjon og kunne bruke straffelovens nødrettsprinsipper eller øyeblikkelig hjelp-plikten i helsepersonelloven. De beskrev at det ble brannslukking heller enn gode løp eller forebyggende tiltak. En deltaker fremhevet også at det fremsto som uklart hvor langt det måtte ha gått før man kunne gripe inn.

Hvis man skal følge regelverket veldig til punkt og prikke, så blir det som at, nei vi kan ikke gripe inn så bruker må bare fortsette å skade seg selv en stund til, til det har blitt så ille at vi kan si at nå er det nødrettssituasjon, nå må vi gripe inn. (Deltaker 6, kommune).

Noen deltakere fremhevet sikkerhetsproblematikk som deres hovedutfordring. Personer som ble beskrevet som voldelige, farlige, og kanskje hadde svært store utfordringer i måten de var på i forhold til andre. Deltakerne fremhevet at de ikke hadde verktøyene for å ha farlige personer i kommunen, og at de ikke ville klare å ivareta disse personene med dagens tjenestetilbud. En deltaker beskrev en økning i antallet personer med slike utfordringer.

De som kommer fra sikkerhet for eksempel, de som kommer fra låste dører. Og noen har vedtak på at de ikke har lov å ha kontakt med omverdenen også. Det er jo mange som opererer på nettet også, og PST er inne i bildet. Disse farligste som vi ikke har noe lovverk for å ha i kommunen. (Deltaker 11, kommune)

I forlengelsen av at enkelte tjenestemottakere kunne ha en utfordrende atferd og/eller være farlige, var det også flere deltakere som fortalte om utfordringer knyttet til ansattes arbeidsmiljø og arbeidsmiljøloven. Som ledere opplevde de det som vanskelig å sikre en trygg og forsvarlig arbeidsplass samtidig som man sikret forsvarlig helsehjelp. Forholdet mellom arbeidsmiljøloven og helselovgivningen opplevdes uklart, og en deltaker beskrev at ingen av lovene hadde noen forrang foran andre. En deltaker lurte på om man egentlig kunne be ansatte om å levere en tjeneste hvis man ikke fulgte arbeidsmiljøloven.

Som ansatt, så har vi en arbeidsmiljølov, og det er der jeg ser utfordringene ofte er. Vi er pliktet til å gi helsehjelp, samtidig er vi egentlig pliktet til å… At mine ansatte skal ha en trygg arbeidsplass. Ikke bli syke eller utsatt for skade. (Deltaker 8, kommune)

Deltakernes bekymringer for arbeidsmiljø knyttet seg til svært dårlig inneklima i private boliger, utskjelling og trusler, samt bruk av vold, og flere deltakere mente at de ansatte måtte tåle for mye. «Folk som jobber der tåler egentlig ganske mye altså. Det gjør de. Som de strengt tatt ikke skulle» (Deltaker 13, statsforvalter). Deltakerne fra en kommune fortalte om erfaring med at en tjenestemottakers levemåte og rusmåte jevnlig medførte at ansatte måtte gå med sikkerhetsutstyr på jobb. En deltaker lurte på om det bare gikk «på lykke og fromme» at det ikke hadde skjedd alvorlige hendelser i deres kommune. Noen stilte spørsmålstegn ved om man kunne trekke seg ut, altså avslutte eller trappe ned tjenestene, når arbeidsmiljøet var dårlig.

### En opplevelse av et komplisert og sammensatt regelverk

Deltakerne ga uttrykk for å ha omfattende kunnskap om de praktiske virkningene av det relevante regelverket. Flere av deltakerne svarte tidlig i intervjuene at regelverket ikke egentlig var så komplisert, og ga uttrykk for at deler av regelverket fungerte relativt greit. Samtidig ga flere uttrykk for frustrasjoner rundt kompleksiteter eller uklarheter i enkelte deler av regelverket. Flere av deltakerne, særlig fra statsforvalterne, beskrev også et komplisert og sammensatt regelverk, som krevde at man hadde kjennskap til helheten. En statsforvalter poengterte at lederne i kommunen nok hadde bedre kunnskap om regelverket enn de som jobbet ute i tjenestene.

Det er ingen plass hvor det egentlig står noe enkelt for folk ute i tjenestene, hvilket regelverk skal vi forholde oss til, fordi det er ingen egne regelverk på dette. Så du må forholde deg til hele regelverket, og det er ekstremt krevende for ikke-jurister. Det er også krevende for advokater. Advokater har ikke peiling i disse sakene som oftest når de involverer seg. Og det sier litt. (Deltaker 5, statsforvalter).

Flere deltakere fra statsforvalterne trakk frem utfordringer knyttet til skjæringspunktet mellom spesialisthelsetjenesten og kommunehelsetjenesten, og fremholdt at det var utfordrende for et godt samarbeid at ansatte i kommunen og spesialisthelsetjenesten hadde begrenset kjennskap til hverandres regelverk.

Området hvor det tilsynelatende rådet mest forvirring blant deltakerne fra både kommune og statsforvalter var knyttet til organisering av botilbud, som ble forutsatt å ha rettslig betydning. Særlig deltakerne fra statsforvalterne beskrev et kjempekomplisert regelverk knyttet til organisering av botilbud, og fremholdt at de trodde det var forvirring rundt dette ute i kommunene. Det ble vist til forvirring rundt grensen mellom bemannet, samlokalisert bolig og institusjon, blant annet fordi institusjonsbegrepet var så vidt i lovgivningen, og en statsforvalter trakk frem mediesaker om besøksbegrensninger på grunn av korona som eksempel. «En har ikke helt visst alltid hvilket regelverk som regulerer det en holder på med» (Deltaker 5, statsforvalter).

Særlig begrepet «omsorgsbolig», og forholdet mellom «omsorgsbolig» og «bofellesskap», «vanlig kommunal bolig», «den der heldøgns-tjenester», «den boligtildelingen etter helselovgivningen» og helse- og omsorgstjenesteloven § 3-2 a, var lite konsekvent mellom deltakerne. «Det er et puslespill, kort og godt» (Deltaker 13, statsforvalter). «Kommunene kaller det jo så forskjellig» (Deltaker 11, kommune). Av noen ble «omsorgsbolig» likestilt med bemannet, samlokalisert bolig, av andre ble det tilsynelatende likestilt med bolig tilrettelagt for heldøgns helse- og omsorgstjenester. «Jeg syns dette er et kjempekomplisert regelverk […] For meg er dette litt uklart faktisk. Jeg får ikke helt tak på det» (Deltaker 9, statsforvalter). Det var også noe variasjon i om deltakerne mente kommunen hadde plikt til å tilby bemannet bolig. Et par deltakere anså boligtilbudet som en del av et samlet tjenestetilbud, og en deltaker mente hjemmelen var kommunens sørge for-ansvar og forsvarlighetskravet, og fremholdt at en bemannet bolig var mer enn at noen var litt nærmere for å gi deg de tjenestene som fulgte av et vedtak. De fleste deltakerne så imidlertid ut til å mene at samlokaliserte, bemannede boliger var en tilleggstjeneste kommunen hadde, eller en måte kommunen valgte å organisere tjenesteytingen på, som falt inn under kommunalt selvstyre.

## Diskusjon

Målet med denne studien var å identifisere utfordringer som tjenesteytere erfarer i det rettslige rammeverket for kommunale bo- og tjenestetilbud til personer med alvorlige ROP-lidelser. Studien har identifisert tre slike utfordringer: ‘en opplevelse av å være avhengig av ‘ekstrainnsats’ ut over lovverkets minimumskrav’, ‘en opplevelse av manglende hjemler og verktøy’, og ‘en opplevelse av et komplisert og sammensatt regelverk’. Vi vil nå drøfte disse utfordringene i lys av statens ansvar for å sikre menneskerettighetene. Vi har også som målsetting å identifisere mulige nye problemstillinger det bør arbeides videre med. Vi vil ikke foreta konkrete rettslige vurderinger, vurdere lovligheten av dagens praksis, eller komme med løsningsforslag til utfordringene. Det må heller gjøres i en annen sammenheng.

Det er lite tidligere forskning på de rettslige rammene for kommunale bo- og tjenestetilbud til personer med alvorlige ROP-lidelser, men det foreligger noe informasjon om hva som mer generelt skal til for å gi gode bo- og tjenestetilbud til gruppen (Helsedirektoratet, 2012, 2014; Røe et al., 2021; Wågø et al., 2019). Denne informasjonen samsvarer med våre deltakeres beskrivelser av hva som skal til for å skape gode tilbud, blant annet er det tidligere vist at tilrettelagte tilbud er hensiktsmessige. I studier av tjeneste*mottakeres* erfaringer med bo- og tjenestetilbud beskrives det også et ønske om differensierte tilbud, og ansatte som er fleksible og tilgjengelige (Andvig & Hummelvoll, 2015; Hansen, 2018). Det som er nytt i vår studie er blant annet at deltakernes beskrivelser har vært knyttet opp mot gjeldende regelverk og at vi dermed tar et nytt perspektiv, et rettslig og menneskerettslig perspektiv, på delvis kjente utfordringer.

### ‘Ekstrainnsats’ og diskrimineringsvern

Den første utfordringen, ‘en opplevelse av å være avhengig av ‘ekstrainnsats’ ut over lovverkets minimumskrav’, viser til vanskeligheter med å få gitt nødvendig hjelp og å nå frem med tjenestene. Utfordringen handler om i hvilken grad lovverket pålegger kommunen plikter, og eventuelt gir tjenestemottakerne tilhørende rettigheter. Det er slik sett ikke et spørsmål om *avveining* av ulike menneskerettigheter, men snarere et spørsmål om hvor langt staten må strekke seg for å sikre personer med alvorlige ROP-lidelser sin rett til tjenester (ØSK, 1966, art. 11, 12). Slik lovverket forstås av de fleste deltakerne, vil et vellykket tilbud i dag være helt avhengig av en omfattende ekstrainnsats fra kommuner og enkeltansatte ut over det lovverket krever. Det kan dermed være grunnlag for å se nærmere på om dagens regelverk sikrer denne gruppen nødvendige eller fungerende tjenester, og/eller om relevante menneskerettighetsinstrumenter oppstiller strengere krav enn det deltakerne erfarte som gjeldende. Staten er forpliktet til å sørge for at det finnes et tilbud av blant annet helsetjenester. ØSK (1966) art. 12 krever at tilbudet blant annet skal være «available», «accessible», «acceptable», og ha en viss kvalitet («quality»), for at det skal anses tilstrekkelig (CESCR, 2000). Slik sett fungerer også de menneskerettslige kravene som minstestandarder, slik nasjonal lovgivning erfares å være av deltakerne i studien. Forutsatt at tilbudet som eksisterer oppfyller de menneskerettslige minimumskravene, er statens plikt til å sikre menneskerettighetene *i utgangspunktet* oppfylt, slik at det er den enkeltes valg å takke ja eller nei, og å velge om man vil benytte seg av sin rett til tjenestene. Samtidig skal personer med alvorlige ROP-lidelser sikres tjenester på lik linje med resten av befolkningen, uten diskriminering (Grunnloven, 1814, § 98; ØSK, 1966, art. 2). Det kan spørres hva diskrimineringsforbudet innebærer overfor personer som er i en spesielt sårbar situasjon, og som i liten grad nyttiggjør seg av tilbud. Krever menneskerettighetene at staten må anstrenge seg ekstra ved å sikre at det tas kreative grep, at man «maser», at man gir ekstra råd og veiledning, og at man har tilpassede boligtilbud, for at man skal kunne påstå at det foreligger et tilstrekkelig tilbud? Det kan være grunn til å se nærmere på om retten til tjenester (ØSK, 1966, art. 11, 12) sammenholdt med diskrimineringsvernet (Grunnloven, 1814, § 98; ØSK, 1966, art. 2) i dette tilfellet innebærer et krav om tilrettelegging av tiltak som muliggjør at personen blir motivert til å motta hjelpen. Det kan her tenkes paralleller til diskusjoner rundt såkalt «Access to Justice» (Frøjd et al., 2022; Kristiansen, 2022) og beslutningsstøtte (Jüriloo, 2016; Østenstad, 2016, 2018).

### Rammer for selvbestemmelse og inngrep

Den andre utfordringen er at deltakerne beskrev at de opplevde å mangle verktøy eller hjemler til å handle. Her befinner man seg i stor grad i den klassiske avveiningen mellom selvbestemmelse på den ene siden, og retten til helse og velferd for personen selv, eller andres rettigheter, på den andre siden. Staten skal samtidig sikre både retten til autonomi og privatliv (EMK, 1950, art. 8), sikre retten til tjenester (ØSK, 1966, art. 11, 12), og sikre andres rettigheter. Av hensyn til personen selv kreves det mer for å sette til side selvbestemmelsen, enn der det gripes inn av hensyn til andre. Dette skillet er «grunnleggende og tidløs» (Søvig, 2007, s. 90). Selvbestemmelsen står særlig sterkt når man foretar valg som kun angår en selv, men når valg også påvirker andre må selvbestemmelsen vike tidligere. Se utførlig om alle de til dels motstridende hensyn og menneskerettigheter som gjør seg gjeldende i en slik avveining hos for eksempel Tvangslovutvalget (NOU 2019: 14). Samtidig reiser dette særlige spørsmål når man taler om ansatte i helse- og omsorgstjenesten. Disse har i denne forstand en dobbeltrolle: de er både borgere som skal beskyttes, samtidig som de er utøvere som handler og sikrer rettigheter på vegne av staten. At det kreves mindre før selvbestemmelsen må vike når det gjelder å beskytte andre, gjelder kanskje ikke i samme grad for tjenesteyter. I noen situasjoner kan det bli et spørsmål om hvor mye helsepersonell skal tåle, og hvor langt man skal strekke seg for å oppfylle sikreplikten overfor tjenestemottakeren. Basert på resultatene i denne studien, kan det være grunnlag for å se nærmere på om helsepersonellets rett til beskyttelse i noen tilfeller skal eller kan medføre innskrenkning i tjenestemottakers *rett til selvbestemmelse og privatliv* (EMK, 1950, art. 8), men også om det kan medføre fullstendig eller delvis innskrenkning i tjenestemottakers *rett til tjenester* (ØSK, 1966, art. 11, 12).

Det er også klare sammenhenger mellom spørsmålet om et krav om tilrettelegging av tjenestene, og spørsmålet om selvbestemmelse og inngrep. Dersom tjenestene tilrettelegges på en slik måte at de når frem til tjenestemottakerne, vil man i større grad få gitt hjelp slik at forverring kan hindres (Helsedirektoratet, 2014), og spørsmålet om inngrep og tvang vil kunne aktualiseres sjeldnere. Et skjerpet krav om tilrettelegging kan altså tenkes å forebygge både forverring hos tjenestemottaker og et opplevd behov for å foreta inngrep hos tjenesteyter, selv om det klart nok ikke vil løse alle situasjoner. Det menneskerettslige kravet til at inngrep må være nødvendige og forholdsmessige (EMK, 1950, art. 8 andre ledd; Østenstad, 2011), medfører allerede på andre områder at man som hovedregel skal ha prøvd frivillige og tillitsskapende tiltak før man utøver tvang. Det er nærliggende å tenke at et tilsvarende krav bør gjelde også når konsekvensene kan være at vedkommende risikerer å få en betydelig forverring i sin livssituasjon.

Som nevnt innledningsvis i resultatdelen var det enkelte statsforvaltere som mente man hadde en utvidet adgang til bruk av tvang og rettighetsbegrensinger, basert på utradisjonelle tolkninger av gjeldende regelverk, ved bruk av analogier og lignende. En aktuell menneskerettslig problemstilling, basert på dette, er i hvilken grad retten til selvbestemmelse og privatliv (EMK, 1950, art. 8) åpner for tvang eller andre rettighetsbegrensinger basert på ulovfestede grunnlag som den alminnelige forvaltningsrettslige vilkårslæren eller såkalt ‘preventiv nødrett/nødverge’. Se for eksempel diskusjonene rundt Sårstelldommen, hvor juridiske forfattere med ulike innfallsvinkler har analysert og kritisert Høyesterett etter at flertallet fant at et sykehjem kunne sette vilkår om gjennomføring av nødvendig sårstell og pleie overfor en samtykkekompetent pasient, og at tiltakene kunne gjennomføres ved tvang (Andersen & Wallevik, 2011; Kjellevold & Aasen, 2011; Østenstad, 2014; Syse, 2011).

### Rettssikkerhet ved komplekse eller uklare regelverk

Den tredje og siste hovedutfordringen som resultatene viser, er knyttet til kompleksitet i regelverket. Når staten er forpliktet til å sikre menneskerettighetene, kan det være en rimelig forståelse av denne plikten at staten må sørge for at rettsregler som finnes er så tydelige at det er mulig å anvende dem, og å sikre tilstrekkelig opplæring av de som handler på vegne av staten. Det hjelper lite om reglene i seg selv er utformet på en måte som er i overenstemmelse med menneskerettighetene, hvis de ikke praktiseres slik.

I denne studien var de største uklarhetene tilsynelatende knyttet til ulike måter å organisere tjenestene på, og det rettslige rammeverket ble oppfattet som uklart av både kommunene selv og av statsforvalterne, som skal fungere som kontrollorgan (pasient- og brukerrettighetsloven, 1999, kapittel 7). En mulig konsekvens er at tjenestemottaker i realiteten ikke får en rettighet som var tiltenkt av lovgiver, fordi tjenesteyter ikke oppfatter dette som en eksisterende rettighet, eller misforstår vilkårene for tildeling. Det kan da stilles spørsmål ved om gruppens rett til tjenester (ØSK, 1966, art. 11, 12) er sikret i tilstrekkelig grad. Om man rettslig sett befinner seg i én organisatorisk form eller en annen, altså i ett regelsett eller et annet, *kan* også ha rettslige konsekvenser i ulike retninger. Når organisasjonsform har betydning for statens mulighet til å foreta rettighetsbegrensninger, slik som ved skillet mellom privat bolig og institusjon (helse- og omsorgstjenesteloven, 2011, kapittel 9, kapittel 10; husleieloven, 1999, § 1-1; NOU 2019: 14; pasient- og brukerrettighetsloven, 1999, kapittel 4) kan uklare regler også indirekte medføre en større innskrenkning i selvbestemmelsen og privatliv enn det er rettslig grunnlag for (EMK, 1950, art. 8). Det er grunnlag for å se nærmere på om reglene er så uklare at de åpner for en praksis som ikke er tiltenkt av lovgiver, eller ikke er i overenstemmelse med menneskerettighetene. Denne studien gir en klar oppfordring til å vurdere om det er mulig å foreta klargjøringer eller opprydninger i gjeldende regelverk. Rettsikkerhetsproblematikken med dagens regler er tydelig, når synet på sentrale rettslige spørsmål varierer mellom deltakerne, som har stor innsikt i hvilke problemstillinger som oppstår i den løpende tjenesteytingen til gruppen. Uklare regler som forstås på ulike måter av ulike aktører kan føre til forskjellsbehandling. At likebehandling er en viktig rettsstikkerhetsverdi kommer blant annet til uttrykk i Grunnloven (1814, § 98). Problemet blir ikke mindre av at det er tale om en sårbar gruppe tjenestemottakere som i liten grad nyttiggjør seg av klagemuligheter og andre kontrollfunksjoner, og som har få talspersoner. Likebehandlingsproblemet har her blitt tematisert i ulike sammenhenger, se f.eks. hos Tvangslovutvalget (NOU 2019: 14.

## Studiens begrensninger

Det er i litteraturen stilt spørsmål ved om beskrivelser fra kvalitative intervjuer gjenspeiler den faktiske, praktiske virkelighet (Jerolmack & Khan, 2014; Lamont & Swindler, 2014). Resultatene i denne studien bygger på konkrete intervjusituasjoner. Deltakernes beskrivelser av hvordan de oppfattet regelverket i møte med virkeligheten kan heller ikke uten videre ses som et uttrykk for gjeldende rett. Resultatene er dermed ikke uttrykk for en objektiv sannhet, hverken faktisk eller normativt, men de gir en verdifull indikasjon på hvordan et utvalg personer som jobber relativt tett på tjenestene oppfatter det relevante regelverket i møte med virkeligheten.

Målet med artikkelen har ikke vært å underlegge utfordringene som har kommet frem i studien en inngående rettsvitenskapelig vurdering eller analyse. Forholdet til menneskerettighetene er relativt overfladisk belyst, og resultatene er i begrenset grad gitt kontekst gjennom en redegjørelse for gjeldende rett. Målet med studien har vært å vise og belyse utfordringene som erfares, og å identifisere nye problemstillinger som det bør arbeides videre med, og vi har derfor ikke foretatt konkrete rettslige avveininger eller forsøkt å komme med løsningsforslag.

I studien er det søkt funnet frem til personer i kommuner og hos statsforvaltere som har særlig kunnskap om hvordan det relevante regelverket fungerer i praksis, altså personer som har en særlig kompetanse gjennom erfaring med brukergruppen, tjenestene og aktuelt lovverk. Studien sier dermed lite om den generelle kunnskapen om regelverket hos ansatte i disse instansene. Deltakerne har også gjennomgående rådgiver- og lederroller, og har ikke dag-til-dag kontakt med tjenestemottakere. Det kan altså tenkes å være andre utfordringer som fremstår mer pressende for andre ansatte i kommunene, for eksempel ansatte i samlokaliserte boliger (NAPHA, 2017).

## Konklusjon

Denne studien har identifisert tre utfordringer i rettslig rammeverk for kommunale bo- og tjenestetilbud til personer med alvorlige ROP-lidelser: ‘en opplevelse av å være avhengig av ‘ekstrainnsats’ ut over lovverkets minimumskrav’, ‘en opplevelse av manglende hjemler og verktøy’, og ‘en opplevelse av et komplisert og sammensatt regelverk’. Disse utfordringene er kort drøftet i lys av grunnleggende menneskerettighetsbestemmelser, hvor det er identifisert flere nye problemstillinger som med fordel kan danne grunnlag for videre forskning. Etter vårt syn er alle de tre identifiserte utfordringene sentrale funn, og omhandler tematikk som kan ha stor betydning for personer med ROP-lidelser. De nye problemstillingene som må vurderes dersom man skal forsøke å ta på alvor utfordringene som kommer frem i studien, dreier seg om tre litt ulike menneskerettighetsperspektiv. For det første, en vurdering av om *staten er forpliktet* til å bruke mer ressurser på å sikre retten til tjenester, *uten* at dette trenger å avveies mot andre rettigheter som selvbestemmelse eller privatliv. For det andre, om man *må eller kan* gå lenger i å sikre retten til tjenester og/eller andres rettigheter *på bekostning* av tjenestemottakerens rettigheter, og altså revurdere balansen mellom ulike menneskerettigheter som kan komme i konflikt. Og for det tredje, å søke å tydeliggjøre gjeldende regelverk i en mer teknisk, men likevel krevende, øvelse, for å sikre de ulike rettighetene.

Løsningene på de skisserte utfordringene og nye problemstillingene er imidlertid ikke gitt. Som nevnt er det ikke i denne artikkelen tatt stilling til hvorvidt menneskerettighetene oppstiller krav som nødvendiggjør endringer. Det *kan* være tale om å korrigere hvordan praksis tolker gjeldende lovverk, for eksempel gjennom utgivelse av nye veiledere eller lignende, eller eventuelt å endre eller lage nytt lovverk. Samtidig er det ikke nødvendigvis slik at endringer eller tilpasninger i rettslig rammeverk er den eneste ‘rette medisin’ for å løse utfordringene. Løsningene kan være andre virkemidler enn rettslige, eller en kombinasjon mellom ulike virkemidler, for eksempel økte budsjetter med eller uten en skjerping av en rettslig aktivitetsplikt. Forfatterne mener ikke at utfordringene som her er skissert utelukkende er rettslige. Dette er spørsmål som også handler om verdivalg, og uavhengig av en klarlegging av de rettslige rammer, er det naturligvis ingenting i veien for at noen av utfordringene søkes løst gjennom for eksempel økte budsjetter og økte kompetansekrav. Og dersom menneskerettighetene ikke oppstiller skjerpede krav, er et al.ternativ også å beholde status quo, dersom man mener at beste løsning i et bredere helhetsperspektiv allerede er nådd. Men ut fra studiens resultater bør flere av de konkrete utfordringene og problemstillingene som her er skissert underlegges en nærmere, rettslig behandling, det bør vurderes hvilke krav menneskerettighetene konkret oppstiller, og det bør vurderes nærmere om rettslig rammeverk i dag hindrer eller ikke i tilstrekkelig grad bidrar til gode tjenestetilbud.
